# Evaluation of choroidal thickness, macular thickness, and aqueous flare after cataract surgery in patients with and without diabetes: a prospective randomized study

**DOI:** 10.1186/s12886-020-01371-7

**Published:** 2020-03-14

**Authors:** Yasuko Ikegami, Miyuki Takahashi, Kana Amino

**Affiliations:** 1 Department of Ophthalmology, Tokyo Metropolitan Geriatric Hospital, 35-2 Sakae-cho, Itabashi district, Tokyo, 173-0015 Japan; 2 Department of Ophthalmology, Graduate School of Medicine, University of Tokyo, Tokyo, Japan; 3 Department of Ophthalmology, School of Medicine, Kitasato University, Kanagawa, Japan; 4 Department of Ophthalmology, Musashino Red Cross Hospital, Tokyo, Japan

**Keywords:** Cataract surgery, Laser flare-cell meter, Retinal thickness, Choroidal thickness, Optical coherence tomography, Diabetic retinopathy

## Abstract

**Background:**

In diabetic eyes, various choroidal abnormalities are noted in addition to changes in the retinal circulation, and the risk of increased aqueous flare and retinal thickening after cataract surgery is higher in diabetic eyes. Inflammation caused by surgery induces breakdown of the blood-retinal barrier and affects the retina, although the influence on the choroid is unknown. Several researchers have evaluated the choroidal thickness (CT) after cataract surgery in patients with diabetes; however, the results are inconsistent. The purpose of this study was to evaluate the influence of uneventful small-incision phacoemulsification cataract surgery on the subfoveal choroidal thickness (SCT), the central macular thickness (CMT), and aqueous flare in patients with diabetes.

**Methods:**

This study included 59 randomly selected eyes (33 eyes of patients with diabetes and 26 eyes of control patients without diabetes) undergoing small-incision cataract surgery. Among the diabetic eyes, 26 were without diabetic retinopathy, and the remaining eyes had non-proliferative diabetic retinopathy. Aqueous flare, CMT, and SCT measurements were performed before and at 1 week, 1 month, and 3 months after surgery.

**Results:**

The postoperative CMT continued to increase significantly until 3 months in both groups. Although the CMT was more in patients with diabetes than in patients without diabetes during the follow-up period, there was no significant difference between the two groups. The aqueous flare value increased until 3 months after surgery in both groups. Although the increase was significant at 3 months after surgery in patients with diabetes, the increase in controls was not significant. The aqueous flare values differed significantly between the two groups before and at 3 months after surgery. There was no significant within-group or between-group difference in pre- and postoperative SCT values.

**Conclusion:**

In diabetic eyes with early stage of retinopathy, even small-incision cataract surgery can induce increased aqueous flare and macular thickening until 3 months, although there is no significant change in the choroidal thickness. Further studies are essential to evaluate choroidal changes after the cataract surgery in diabetic eyes.

## Background

Cataract is the leading cause of visual impairment in the elderly, and typically requires surgical treatment. Cataract surgery is an invasive procedure that releases inflammatory mediators, which diffuse from the anterior chamber to the posterior segment and lead to the breakdown of the blood-aqueous barrier and blood-retinal barrier (BRB) [1]. The aqueous flare value measured by a laser flare-cell meter reflects the degree of inflammation and the function of the blood-aqueous barrier and BRB [2]. Several studies have reported increased flare values after cataract surgery [3, 4]. The breakdown of the BRB results in increased permeability of the perifoveal capillaries with fluid accumulation in the retina, causing cystoid macular edema (CME) to develop [1, 5]. It is widely known that the macular thickness increases after cataract surgery, which could lead to CME [6–9]. The macular thickness can be measured using optical coherence tomography (OCT), and some studies have reported increased aqueous flare values in patients with CME after cataract surgery [10]. Recently, with technical improvements in cataract surgery, such as smaller incisions and shorter surgeries, surgical inflammation and the degree of BRB breakdown are reduced. However, there are few reports of aqueous flare and the macular thickness after uneventful minimally invasive cataract surgery.

Diabetes mellitus is a vascular disease characterized by macrovascular and microvascular abnormalities. It is reported that the risk of a higher aqueous flare value [11] and macular thickening [12–17] after uncomplicated phacoemulsification is increased in patients with diabetes.

The choroid is the highly vascularized layer of the eye. Per unit weight, the choroid has the highest blood flow of any tissue in the body and is the vascular supply to the outer retina and retinal pigment epithelium (RPE), and it is the only source of metabolic exchange for the avascular fovea [18]. With the assistance of Enhanced Depth Imaging or Swept Source OCT, several researchers have evaluated the choroidal thickness (CT) after cataract surgery [19–23]; however, the results are conflicting.

In the eyes of patients with diabetes, in addition to changes in retinal circulation, various choroidal abnormalities were noted in histopathological studies. The findings included abnormalities such as arteriosclerosis, obstruction of the choriocapillaris, vascular degeneration, focal scarring, increased tortuosity of vessels, focal vascular dilatation and narrowing, neovascularization, aneurysm, and deficits in the choroidal vasculature [24, 25]. Recently, several studies have investigated CT in patients with diabetes. Most groups reported reduced CT in patients with diabetic retinopathy [26–29], and several groups reported that the choroid was thinner in eyes with diabetic macular edema (DME) [30–32]. Although some groups have reported CT after cataract surgery in patients with diabetes [33–35], the results have been inconsistent.

Therefore, the purpose of this study was to examine the changes in aqueous flare, central macular thickness (CMT), and subfoveal choroidal thickness (SCT) before and after minimally invasive cataract surgery, and to evaluate the influence of diabetes on these changes.

## Methods

### Patients and study design

Patients undergoing cataract surgery between March 2016 and June 2017 were divided into two groups: patients with diabetes and patients without diabetes, and randomly selected for enrollment. The inclusion criteria were: cataract in one or both eyes with adequate OCT linear scans. The exclusion criteria were: axial length of > 26.5 mm or < 21 mm in the affected eye; significant media opacities; aged < 50 and > 90 years; diagnosis or history of any ocular diseases that might influence the study results such as glaucoma, uveitis, age-related macular degeneration, retinal vaso-occlusive disease, and neurodegenerative disease; and refusal to provide informed consent. At enrollment, a complete ophthalmologic examination and five-field fundus photography were performed.

This study was approved by the institutional ethics committee (No: 27022), and written informed consent was obtained from all patients after adequate study explanation. All research and measurements adhered to the tenets of the Declaration of Helsinki.

### Aqueous flare measurement

The aqueous flare intensity was measured using a laser flare-cell meter (FC-2000 Kowa Co, Nagoya, Japan). Seven consecutive flare readings with background scatter of < 15% were acquired for each eye and averaged after excluding the minimum and maximum measurements in each series of readings. The results are expressed as photon counts per millisecond (pc/ms). The flare intensity was measured 30–60 min after the application of 0.5% tropicamide and 0.5% phenylephrine hydrochloride eye drops.

### Spectral-domain OCT

Optical coherence tomograms were acquired through dilated pupils using spectral domain (SD) OCT (Spectralis, Heidelberg Engineering Co, Heidelberg, Germany). The OCT imaging technique involved obtaining a macular square (20 × 20°) composed of 25 horizontal parallel B-scans. CMT is defined as the mean thickness within the central 1 mm diameter area in the fast macular thickness map obtained by automated measurement.

Additionally, for each case, a single horizontal and vertical B-scan, averaged 100 times, centered on the fovea was obtained. All scans were performed in enhanced depth imaging mode to improve the quality of choroidal imaging. The SCT was measured manually on the single horizontal and vertical B-scan using the caliper found on the Spectralis software. Two masked, independent observers measured the SCT perpendicularly from the outer limit of the subfoveal RPE to the inner scleral boundary. The measurements from the two observers were then averaged for analysis. Postoperative OCT images were acquired using the “follow-up” function of Spectralis to ensure that the same retinal and choroidal areas were scanned.

### Cataract surgery

All included cases were submitted to regular phacoemulsification cataract surgery with intraocular lens implantation in the capsular bag. All surgeries were performed by two highly experienced ophthalmologists. No patients experienced surgical complications. Phacoemulsification surgery was performed via a 2.8 mm incision in the temporal clear cornea. A silicone intraocular lens (KS-1, AQ-110NV, Staar, Japan) or acrylic intraocular lens (Avansee PU-6, Kowa, Japan) was implanted. Postoperative medication consisted of 0.1% betamethasone, 0.5% moxifloxacin hydrochloride, and 0.1% diclofenac sodium eye drops for 4 weeks, followed by 0.1% diclofenac sodium eye drops for an additional 2 months.

### Patient assessment

OCT and laser flare-cell meter measurements were performed immediately before cataract surgery and 1 week, 1 month, and 3 months after cataract surgery. All measurements were performed between 11:00 and 15:00 to avoid the effects of diurnal variation.

### Statistical analysis

Changes in the CMT, SCT, and aqueous flare values before and after surgery were analyzed by a repeated-measures ANOVA or paired sample *t*-test with a Bonferroni post-test. The data are expressed as the mean ± standard deviation. Normally distributed data were analyzed with the Kolmogorov-Smirnov test. A *P* value of < 0.05 was considered statistically significant. The *P* values that remained significant after Bonferroni correction for multiple comparisons were adopted. Statistical analyses were performed using EZR (Saitama Medical Center, Jichi Medical University, Saitama, Japan), which is a graphical user interface for R (The R Foundation for Statistical Computing, Vienna, Austria) [36].

## Results

This prospective study included 59 eyes of 45 patients (24 women and 21 men) with a mean age of 74.7 ± 7.8 years (range, 58–90 years). The study included 33 eyes of 23 patients with diabetes (12 women and 11 men) and 26 eyes of 22 patients without diabetes (12 women and 10 men). All patients with diabetes had type 2 diabetes mellitus. The mean age of the patients with and without diabetes was 76.21 ± 7.21 years and 72.73 ± 8.06 years, respectively. Before surgery, the mean axial length was 22.97 ± 1.10 mm and 23.40 ± 1.28 mm in the patients with and without diabetes, respectively. There were no significant differences in age (*P* = 0.09), sex (*P* = 0.79), or axial length (*P* = 0.18) between the diabetic and non-diabetic groups (Table 1).

**Table 1:** Baseline demographic of the enrolled eyes

	Diabetic eyes	Non-diabetic eyes	*P* Value
Eyes (patients)	33 (23)	26 (22)	
Age (mean ± SD)	76.21 ± 7.21	72.73 ± 8.06	0.09
Male/Female	11/12	10/12	0.79
Axial Length (mean ± SD)	22.97 ± 1.10	23.40 ± 1.28	0.18
HbA1c, % (mean ± SD)	6.95 ± 0.76	–	

Among the patients with diabetes, two were treated with insulin, 20 with oral hypoglycemic drugs, and one with only diet therapy. The mean HbA1c level was 6.95 ± 0.76%. Each eye underwent five-field fundus photography before surgery, and the severity of diabetic retinopathy was assessed on fundus findings and classified according to the International Clinical Diabetic Retinopathy Disease Severity Scale published in 2002 [37]. Twenty-six eyes did not exhibit any signs of diabetic retinopathy, four eyes had mild non-proliferative diabetic retinopathy (NPDR), two eyes had moderate NPDR, and one eye had severe NPDR. CME was not observed by OCT in any patients during follow-up.

**Fig. 1:**
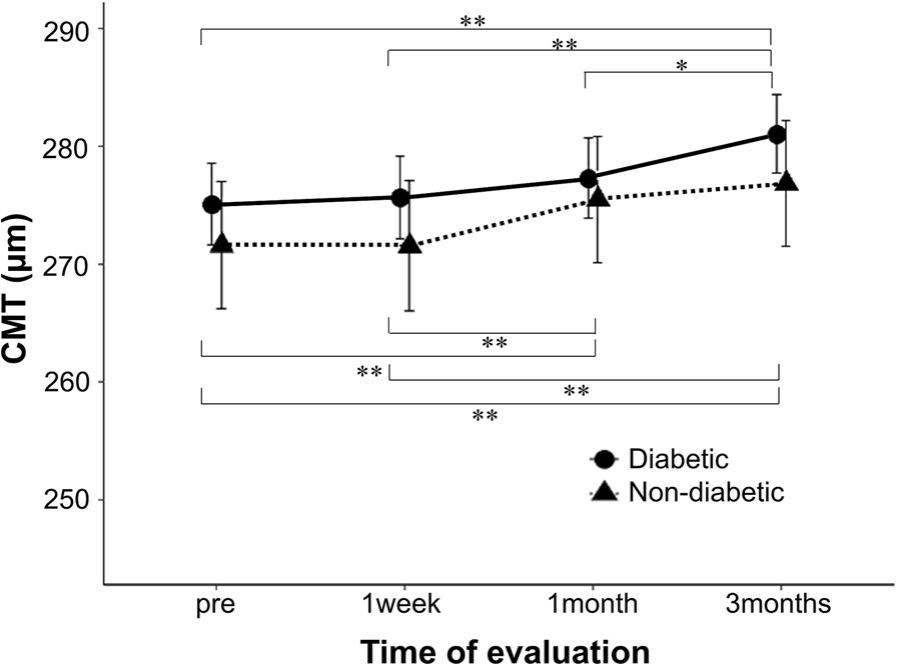
Changes in the central macular thickness (CMT) before (“pre”) and after cataract surgery. Comparison of CMT between diabetic and non-diabetic eyes. The asterisks (*) indicate a statistically significant difference on paired sample *t*-test with Bonferroni post-test (*P < 0.05, **P < 0.01). The vertical bars represent ±1 unit of standard error of mean

The mean preoperative CMT was 275.09 ± 19.75 μm and 271.62 ± 27.00 μm in patients with and without diabetes, respectively. The postoperative CMT increased during follow-up in both groups, and after Bonferroni correction, there was a significant increase compared to the preoperative value at 3 months after surgery in patients with diabetes, and at 1 and 3 months after surgery in patients without diabetes. The CMT values tended to be higher in patients with diabetes than in those without diabetes; however, there were no significant differences between the groups (Fig. 1; Table 2).

**Table 2:** Aqueous flare, CMT and SCT before(“pre”) and after cataract surgery

		Diabetic eyes	Non-diabeic eyes	*P* value
Aqueous flare, pc/ms (mean ± SD)	pre	7.42 ± 3.29	5.57 ± 2.39	0.021*
	1 week	8.08 ± 4.31	6.97 ± 2.28	0.215
	1 month	8.03 ± 3.55	6.90 ± 2.77	0.194
	3 months	9.67 ± 5.32	7.19 ± 2.82	0.028*
CMT, μm (mean ± SD)	pre	275.09 ± 19.75	271.62 ± 27.00	0.577
	1 week	275.64 ± 19.77	271.54 ± 27.58	0.517
	1 month	277.30 ± 19.23	275.50 ± 26.74	0.768
	3 months	281.06 ± 18.98	276.85 ± 26.93	0.492
SCT, μm (mean ± SD)	pre	222.05 ± 96.73	234.27 ± 71.42	0.599
	1 week	224.38 ± 99.11	236.96 ± 74.88	0.599
	1 month	226.12 ± 99.39	236.88 ± 74.04	0.653
	3 months	226.23 ± 101.60	242.35 ± 73.79	0.507

*Statistically significant *P* value

**Fig. 2:**
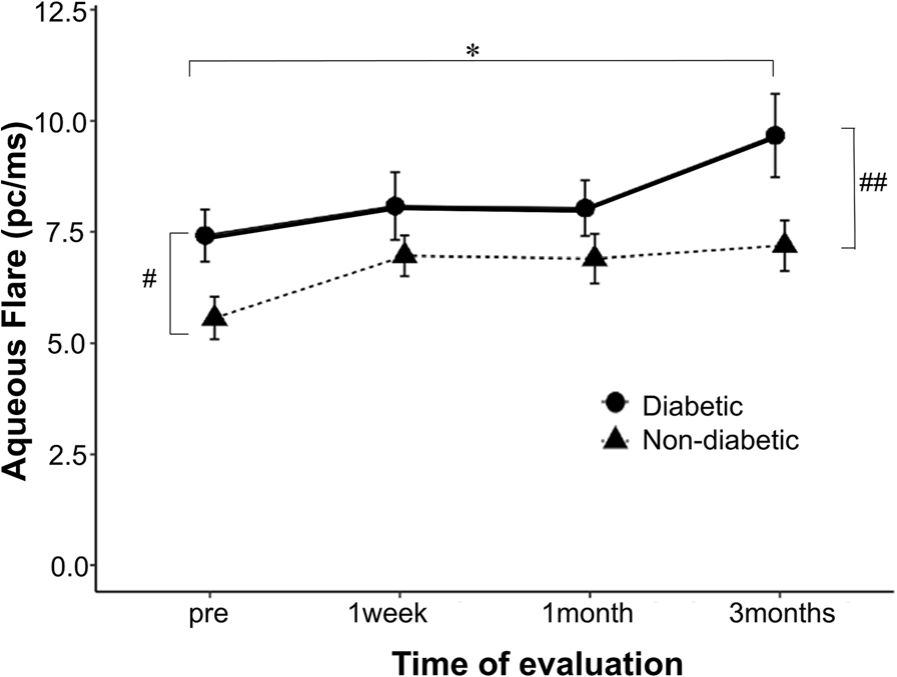
Changes in aqueous flare values before (“pre”) and after cataract surgery Comparison of the aqueous flare values between diabetic and non-diabetic eyes. The asterisks (*) indicate statistically significant differences on paired sample *t*-test with Bonferroni post-test (*P < 0.05). Sharps (#) indicate statistically significant differences (#*P* < 0.05, ##*P* < 0.01; *t*-test). The vertical bars represent ±1 unit of standard error of mean

The aqueous flare value before surgery was 7.42 ± 3.29 pc/ms and 5.57 ± 2.39 pc/ms in patients with and without diabetes, respectively, and the difference was significant. The aqueous flare value after surgery was increased compared to the preoperative value in both groups, and there was a significant increase at 3 months in patients with diabetes. The aqueous flare values differed significantly between the groups before surgery and at 3 months after surgery (Fig. 2; Table 2).

**Fig. 3:**
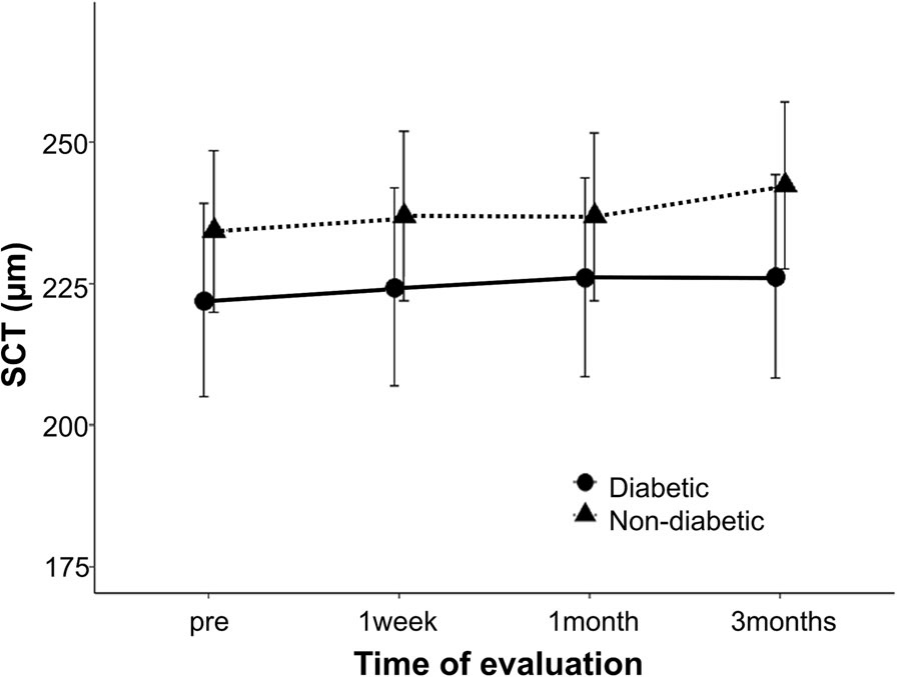
Changes in the subfoveal choroidal thickness (SCT) before (“pre”) and after cataract surgery**.** Comparison of SCT between diabetic and non-diabetic eyes. The vertical bars represent ±1 unit of standard error of mean

The mean preoperative SCT was 222.05 ± 96.73 μm and 234.27 ± 71.42 μm in patients with and without diabetes, respectively. The postoperative SCT increased compared to the preoperative value throughout the follow-up period in both groups; however, after Bonferroni correction, there was no significant difference between the preoperative and postoperative values (Fig. 3). The SCT values tended to be lower in patients with diabetes than in those without diabetes; however, there were no significant differences between the two groups (Table 2).

## Discussion

The results of the present study clearly demonstrated a significant increase in the CMT until 3 months after cataract surgery in both diabetic and non-diabetic eyes. Moreover, there was a significant increase in the aqueous flare value after cataract surgery in diabetic eyes; this confirmed the findings of previous reports [3, 7, 8]. A recent large real-world study indicated that the incidence of CME peaked at 3–6 months after surgery in patients with diabetes; this finding is nearly consistent with our finding, although previous studies have reported that CME developed within 4–6 weeks after cataract surgery in most cases [16, 17]. From these findings, we reconfirmed persistent postoperative inflammatory activity even in minimally invasive surgery with a small incision performed by experienced surgeons.

The aqueous flare values were significantly higher in the patients with diabetes than in those without diabetes during the follow-up period, and the CMT values tended to be higher in patients with diabetes than in those without diabetes; however, the difference was not significant. As previously reported [17], antiphlogistic eye drops are considered useful and necessary for reducing postoperative inflammation in the anterior chamber, particularly in patients with diabetes. Some researchers stated that, in patients with diabetes, the incidence of CME after cataract surgery, even in the absence of diabetic retinopathy, is more frequent because the BRB is already damaged before surgery [14]. Several reports suggested that, in diabetic eyes, the elevated levels of various inflammatory mediators in the aqueous humor may play a role in the breakdown of the BRB [38, 39]. However, in the present study, the effect of increased aqueous flare on the retina was considerably small. Minimally invasive, uncomplicated surgery conducted by an experienced surgeon induced minimal changes in the BRB, and diabetes had almost no effect on the retina. We also found that the baseline CMT value was not significantly different between the diabetic and non-diabetic groups. This indicates that the effect of diabetes on the retina and BRB was not apparent from the beginning. In the retina that is minimally affected by diabetes, the influence of surgery may be similar to that in the retina without diabetes. Furthermore, the mean HbA1c level of patients was 6.95% ± 0.76%, and diabetes was relatively well controlled with mild or no retinopathy in all patients. These factors may have resulted in the lack of significant differences in the CMT between the two groups.

The main finding of our study was the lack of a significant difference in CT after cataract surgery in both groups and between diabetic eyes and non-diabetic eyes during the follow-up period. Our findings confirmed the correlation between CT and cataract surgery reported by Brito et al. [34] and Falcão et al. [23] In these studies, cataract surgery did not induce choroidal changes, and the CT was free of the inflammation of the anterior chamber and inner retina caused by BRB disturbance. The absence of change in CT might be due to the fact that the retinal capillaries have a BRB and are autoregulated, whereas the choroidal capillaries are not autoregulated and thus behave differently from the retinal vessels.

Although there are many reports on CT in diabetic patients, the effect of diabetes on the choroid remains to be established [26–29, 31]. The general consensus is that diabetes causes a reduction in CT. Some authors have suggested that reduced CT in diabetes is due to the loss or drop out of the choriocapillaris, relative vasoconstriction, and reduced blood flow into the choriocapillaris [26]. Choroidal vessels provide nutrients to the RPE and outer retinal layers, and the reduced CT at the fovea, accompanied by retinal hypoxia, may cause the onset of diabetic retinopathy [40]. Retinal hypoxia increases vascular endothelial growth factor expression in RPE, pericytes, and microvascular endothelial cells, and may result in breakdown of the BRB, which induces diabetic retinopathy or maculopathy [27, 41, 42].

We revealed that while the SCT tended to be thinner in patients with diabetes than in those without diabetes throughout the follow-up period, the difference was not statistically significant. A possible reason for this result is that the eyes included in this study had no retinopathy or were in the early stage of retinopathy. In the early stage of diabetes, the diabetic angiopathic changes tend to be less severe in the choroidal capillaries and therefore cannot be detected by SD-OCT [34]. Lee et al. suggested that the functional integrity of the choroid in diabetic retinopathy is preserved after initial damage because the choroid is relatively resistant to the effects of diabetic retinopathy owing to a surplus of choroidal vessels and sufficient blood flow [43].

Odrobina et al. reported that the SCT in eyes with postoperative CME was significantly thinner than that in fellow eyes without CME [32]. Moreover, other researchers have reported that the choroid is also thinner in eyes with DME than in eyes without DME [36, 44]. Odrobina et al. speculated that the mechanisms may be similar to those in diabetic retinopathy. Since there is no retinal vasculature in the foveal region, impairment of the choriocapillaris may cause severe functional damage to the retinal tissue in the fovea.

In this study, none of the patients developed CME during the follow-up period, and the CMT increase was relatively small. The CMT values tended to be higher in patients with diabetes than in those without diabetes; however, the difference was not significant. Furthermore, 26 of 33 eyes of patients with diabetes did not exhibit retinopathy. In addition, the sample size of this study was relatively small. These factors may explain why no significant changes were observed in the SCT before and after surgery. We considered that in patients with diabetes whose retinopathies were in the relatively early stage, cataract surgery did not affect the SCT and the choroidal influence on the retina was undetectable.

This study had limitations such as a short follow-up period and a relatively small sample size. There were also measurement errors in the analysis of the SCT, as the SCT measurements were performed manually. In addition, we analyzed only the mean SCT and mean CMT, leading to a lack of segmentation of these parameters. There is great variability even in the SCT of healthy eyes. Studies of healthy eyes have revealed variations in CT with age, axial length, and even time of day, with diurnal variation in the SCT [45]. We excluded the eyes with the shortest and longest axial lengths, and all examinations were performed at almost the same time of day; however, we could not exclude all of the parameters that may affect SCT measurement.

## Conclusions

In this study, we examined three parameters: aqueous flare, CMT, and SCT, simultaneously. To the best of our knowledge, this is the first study to measure these three parameters simultaneously at different time points before and after cataract surgery. This report suggested that in diabetic eyes, minimally invasive cataract surgery could induce increased inflammation as indicated by the aqueous flare, which was accompanied by an increase in macular thickness. Therefore, more careful examination is required in diabetic eyes after cataract surgery. Although the postoperative aqueous flare differed significantly between diabetic and non-diabetic eyes, the differences in the postoperative CMT and SCT were not significant between the two groups. One possible reason is that the eyes included in our study were in a relatively early stage of retinopathy. Further studies with a greater number of patients, longer follow-up duration, and eyes in a more advanced stage of retinopathy are needed.

## Data Availability

The datasets used and analyzed during the current study are available from the corresponding author on reasonable request.

## Abbreviations

BRB: Blood-retinal barrier
CME: Cystoid macular edema
CMT: Central macular thickness
CT: Choroidal thickness
DME: Diabetic macular edema
NPDR: Non-proliferative diabetic retinopathy
OCT: Optical coherence tomography
RPE: Retinal pigment epithelium
SD-OCT: Spectral domain OCT
SCT: Subfoveal choroidal thickness
